# A Bacterial Pathogen Displaying Temperature-Enhanced Virulence of the Microalga *Emiliania huxleyi*

**DOI:** 10.3389/fmicb.2016.00892

**Published:** 2016-06-13

**Authors:** Teaghan J. Mayers, Anna R. Bramucci, Kurt M. Yakimovich, Rebecca J. Case

**Affiliations:** Department of Biological Sciences, University of Alberta, EdmontonAB, Canada

**Keywords:** *Emiliania huxleyi*, *Ruegeria*, roseobacter, ocean warming, marine pathogen, temperature-enhanced virulence, climate change, phytoplankton

## Abstract

*Emiliania huxleyi* is a globally abundant microalga that plays a significant role in biogeochemical cycles. Over the next century, sea surface temperatures are predicted to increase drastically, which will likely have significant effects on the survival and ecology of *E. huxleyi*. In a warming ocean, this microalga may become increasingly vulnerable to pathogens, particularly those with temperature-dependent virulence. *Ruegeria* is a genus of Rhodobacteraceae whose population size tracks that of *E. huxleyi* throughout the alga’s bloom–bust lifecycle. A representative of this genus, *Ruegeria* sp. R11, is known to cause bleaching disease in a red macroalga at elevated temperatures. To investigate if the pathogenicity of R11 extends to microalgae, it was co-cultured with several cell types of *E. huxleyi* near the alga’s optimum (18°C), and at an elevated temperature (25°C) known to induce virulence in R11. The algal populations were monitored using flow cytometry and pulse-amplitude modulated fluorometry. Cultures of algae without bacteria remained healthy at 18°C, but lower cell counts in control cultures at 25°C indicated some stress at the elevated temperature. Both the C (coccolith-bearing) and S (scale-bearing swarming) cell types of *E. huxleyi* experienced a rapid decline resulting in apparent death when co-cultured with R11 at 25°C, but had no effect on N (naked) cell type at either temperature. R11 had no initial negative impact on C and S type *E. huxleyi* population size or health at 18°C, but caused death in older co-cultures. This differential effect of R11 on its host at 18 and 25°C suggest it is a temperature-enhanced opportunistic pathogen of *E. huxleyi.* We also detected caspase-like activity in dying C type cells co-cultured with R11, which suggests that programmed cell death plays a role in the death of *E. huxleyi* triggered by R11 – a mechanism induced by viruses (EhVs) and implicated in *E. huxleyi* bloom collapse. Given that *E. huxleyi* has recently been shown to have acquired resistance against EhVs at elevated temperature, bacterial pathogens with temperature-dependent virulence, such as R11, may become much more important in the ecology of *E. huxleyi* in a warming climate.

## Introduction

Ocean warming is one of the largest contemporary threats to the stability of the marine ecosystem. Since the 1990s, the average global sea surface temperature (SST) has been reported to be rising – an increase as dramatic as 4°C in some regions – and the rate of increase continues to climb (Smith et al., 2008). In the last century, there has been a global decline in phytoplankton which is strongly correlated to increasing SST (Boyce et al., 2010). This trend is disturbing, given the fact that phytoplankton form the base of the marine food web, and account for over half of the earth’s primary productivity annually (Behrenfeld et al., 2001).

*Emiliania huxleyi* (Prymnesiophyta) is a ubiquitous marine phytoplankton and is the most common representative of the extant coccolithophores (Paasche, 2002). The life cycle of *E. huxleyi* alternates between diploid C type cells (non-motile coccolith bearing) and haploid S type cells (flagellated with organic scales), both of which are capable of propagating indefinitely via mitosis (Paasche, 2002). There is indirect evidence that S cells are generated through meiosis of C cells, and conversely, C cells are regenerated through syngamy of S cells; however, neither of these processes has been directly observed (Green et al., 1996; Paasche, 2002; von Dassow et al., 2009). A third, less common cell type, also exists – diploid non-motile N (naked) cells. These are thought to be naturally occurring mutants of C cells lacking the ability to produce coccoliths (Paasche, 2002; Frada et al., 2012).

*Emiliania huxleyi* is a model organism and has been studied extensively due to its significant role in global biogeochemical cycles (Simó, 2004). As a major producer of dimethylsulfoniopropionate (DMSP), the most abundant source of organic sulfur in the ocean, *E. huxleyi* is hypothesized to play a role in regulating earth’s climate (Charlson et al., 1987; Ayers and Cainey, 2007). Under conditions that promote increased growth in *E. huxleyi* (increased temperature, increased availability of carbon for photosynthesis, etc.), DMSP production is increased (Ayers and Cainey, 2007). DMSP is degraded to dimethyl sulfide (DMS) by bacteria and phytoplankton, and this serves as the main contributor of cloud condensation nuclei (CCN) in marine environments (Charlson et al., 1987; Ayers and Cainey, 2007; Reisch et al., 2011). An increase in CCN production may lead to increased cloud cover and a subsequent increase in albedo, which has a cooling effect on atmospheric temperature (Charlson et al., 1987; Ayers and Cainey, 2007). However, this feedback hypothesis is highly debated (Quinn and Bates, 2011). Recent work has shown that the production of DMS by *E. huxleyi* cultures decreases at high CO_2_ concentrations in mesocosm experiments (Webb et al., 2016). This indicates that one of the marine environments temperature regulation mechanisms may be hindered by anthropogenic CO_2_ emissions.

*Emiliania huxleyi* also plays a unique role in carbon cycling. In addition to organic photosynthate production, *E. huxleyi* produces elaborate calcium carbonate disks (coccoliths) that cover its cells. Although the function of these coccoliths remains unclear, they may aid in UV protection (Gao et al., 2009). During the natural senescence (Voss et al., 1998; Chow et al., 2015) and virally induced (Wilson et al., 2002) death of an *E. huxleyi* cell, its coccoliths are shed. These calcite coccoliths are denser than the surrounding seawater, so they sink and are eventually deposited in the deep ocean where they are essentially removed from the carbon cycle (Schmidt et al., 2013). Since *E. huxleyi* displays a lifestyle in which expansive blooms – sometimes hundreds of thousands of square kilometers in size – appear suddenly and unpredictably, then collapse rapidly (Brown and Yoder, 1994), the influence of this phytoplankton on the sulfur and carbon cycles, as well as the proximate biological ecosystem, is maximized during these bloom events. Because of these major roles in global processes, it is essential to understand the ecology of such an influential organism.

*Emiliania huxleyi* lives in close association with a diverse assemblage of microbes (Green et al., 2015). This microbial community is defined by complex and intimate metabolic exchange and communication (Sapp et al., 2007). Some members of this microbial consortium are mutualistic. For example, *E. huxleyi* lacks the ability to synthesize vitamin B12 – a nutrient essential to its growth – however, it has been shown that it is able to survive in culture due to the exogenous production of vitamin B12 by a closely associated bacterium (Helliwell et al., 2011).

Conversely, several microbes associated with *E. huxleyi* are pathogenic. Bloom collapse has been attributed to outbreaks of EhVs – members of the *Phycodnaviridae*, a group of viruses known to infect microalgae (Wilson et al., 2009). The EhVs elicit death in *E. huxleyi* by up-regulating metacaspase activity and causing the associated caspase-like programmed cell death (PCD) of the alga (Bidle et al., 2007). The role of metacaspases and PCD in the defense of land plants to microbial pathogens has been repeatedly characterized (Lam et al., 2001). The same type of response has been suggested for *E. huxleyi* to prevent outbreaks of disease in large clonal populations (Bidle et al., 2007).

The roseobacter clade (α-Proteobacteria) is one of the most abundant bacterial groups present during *E. huxleyi* blooms (second only to SAR lineages; Gonzalez et al., 2000), and contains several pathogenic representatives. One such representative has been demonstrated to be the causative agent in the formation of gall-like tumors on *Prionitis lanceolata* (Rhodophyta; Ashen and Goff, 1998). Additionally, the roseobacter clade also contains the only known bacterial pathogen of *E. huxleyi* – *Phaeobacter gallaeciensis –* that produces potent algaecides in response to *p*-coumaric acid, a senescent signal in plants (Seyedsayamdost et al., 2011).

Another example of an algaecidal roseobacter is *Ruegeria* sp. R11 (syn. *Nautella* sp. R11; Fernandes et al., 2011), which causes bleaching disease in the habitat forming *Delisea pulchra* – a red macroalgae native to the waters surrounding southern Australia (Case et al., 2011). The ability of R11 to cause bleaching events in *D. pulchra* has been demonstrated in both field and laboratory experiments to be temperature-dependent, with the disease only presenting at elevated temperature (Case et al., 2011). R11 was originally isolated from *D. pulchra* in the Tasman Sea (Case et al., 2011), one of the global hot spots for *E. huxleyi* blooms (Brown and Yoder, 1994). The present study aims to test the pathogenicity of R11 on *E. huxleyi*, a ubiquitous microalgae, which blooms in regions overlapping with *D. pulchra*’s geographical distribution (western and southern Australia, and New Zealand; Huisman, 2000), and to assess the role a warming ocean might play in this interaction. Bacterial–macroalgal symbioses have been studied in detail, however, very few bacterial–microalgal interactions have been described (Egan et al., 2013).

It has been predicted that ocean warming will increase the frequency and severity of pathogenic attacks (Harvell et al., 2002). Consequently, it is essential to study the effects of shifting temperature on the biotic interactions of ecologically important organisms, like *E. huxleyi*. In the present study, we demonstrate that R11 is a temperature-enhanced pathogen of both the C and S cell types of *E. huxleyi*, but not N cell type, and that a caspase-like response is induced by R11 in the C cells at elevated temperature, which results in precipitous algal death.

## Materials and Methods

### Growth and Maintenance of Algal and Bacterial Strains

Three axenic strains of *Emiliania huxleyi* were obtained from the Provasoli-Guillard National Centre for Marine Algae and Microbiota (NCMA): a C type diploid coccolith-bearing strain – CCMP3266; an S type haploid sexual strain – CCMP3268; and an N type diploid bald strain – CCMP2090. All strains were maintained in L1-Si media (Guillard and Hargraves, 1993) at 18°C in a diurnal incubator (8:16 h dark–light cycle). Algal cultures and media were checked for bacterial contamination prior to use in experiments by microscopic observations and by inoculation onto ½ marine agar (18.7 g Difco Marine Broth 2216 supplemented 9 g NaCl and 15 g Difco agar in 1 L). All strains were grown statically for 5 days in the 18°C incubator under the same light–dark regimen under which they were maintained. These incubation periods allowed the cultures to reach early-log phase prior to the start of an experiment.

The bacterium, *Ruegeria* sp. R11, was maintained on ½ marine agar at 30°C. It was grown to stationary phase in 5 mL ½ marine broth (18.7 g Difco Marine Broth 2216 supplemented 9 g NaCl) in a shaking incubator (160 rpm) at 21.5°C for 24 h prior to experiments.

### Control Cultures and Co-cultures

For each algal strain tested, control cultures of the algae alone and *Ruegeria* sp. R11 alone, as well as a co-culture of R11 and algae, were prepared as previously described by Bramucci et al. (2015). Briefly, a stationary phase culture of R11 was grown, washed twice by centrifugation and re- in L1-Si media before undergoing a serial dilution in L1-Si to the 10^2^ cfu/mL (cells are diluted to the correct order of magnitude and the exact initial cell concentration is calculated from cfu counts). To prepare the co-culture, an early log-phase culture (5-day-old, 10^4^– 0^5^ cells/mL) was mixed volumetrically 1:1 with the 10^2^ cfu/mL R11. Control cultures of both R11 and the algae were prepared by mixing the respective culture volumetrically 1:1 with sterile L1-Si medium, to account for the ½ dilution of the co-culture. The controls and co-culture were then aliquoted in 1 mL volumes into 48-well microtiter plates (Becton, Dickinson and Company, Franklin Lakes, NJ, USA). Aliquots were dispensed in such a way that three independent replicates of each culture type could be sampled and sacrificed for each time point. This method allowed a time course experiment to be conducted using sacrificial sampling, eliminating the need for re-sampling and reducing the error involved in an experiment with diminishing culture volume (Bramucci et al., 2015).

Half of the microtiter plates were incubated at 18°C, while the other half were incubated at 25°C. The microtiter plates were then incubated statically (8:16 h dark–light cycle) at the respective temperatures for 24 days. This protocol was carried out for each of the three algal strains tested.

### Fluorescence Measurements

A pulse-amplitude-modulation (PAM) fluorometer (WATER-PAM, Waltz, Effeltrich, Germany) was used to measure photosynthetic yield (*F*_v_/*F*_m_) of cultures containing algae (Schreiber et al., 1986). On sampling days, all samples were taken at the mid-point of the dark cycle (at 4 h) and diluted in L1-Si media to within the detection range of the PAM fluorometer. Samples were kept in the dark and at the appropriate temperature (18 or 25°C) throughout sampling. For each sample, an initial dark adaption period of 3 min was administered, after which a saturating pulse was applied and the fluorescence readings were taken twice at intervals of 1 min 30 s to calculate the photosystem II (PSII) potential quantum yield (*F*_v_/*F*_m_) – which indicates the efficiency of PSII (van Kooten and Snel, 1990). Duplicate readings of each sample were averaged and this average was used to determine the *F*_v_/*F*_m_ of each sample (in triplicate). After culture death occurred, artificial yield values were detected for some samples. Severe damage to the chloroplasts and calvin cycle has been shown to result in an artificially high yield (1998), and for this reason yield data were reported as not detectable for samples where both chlorophyll content and cell number indicated that the culture was dead. Data were analyzed using SigmaPlot 12.

### Enumerating Algal and Bacterial Population Density

Algal samples were prepared for flow cytometry from control cultures and co-cultures. Cells were fixed for flow cytometry by incubating in the dark for 10 min with 0.6% glutaraldehyde. Cells were then flash-frozen in liquid nitrogen and stored at -80°C until flow cytometry was performed using a FACSCalibur (Becton Dickinson, San Jose, CA, USA). A single replicate was randomly chosen and analyzed for each experimental day, as experimental variability was low. A 488 nm laser was used for excitation and a 670 nm laser was used for detection of chlorophyll. Chlorophyll autofluorescence was used for cellular enumeration. Cells were subsequently stained with SYBRgreen-I (Life Technologies, Carlsbad, CA, USA) for DNA detection (520 nm). Data were processed using FlowJo 9.2.

The R11 population density from co-culture experiments was enumerated by counting colony forming units (cfu) to enumerate planktonic R11 cells and those attached to *E. huxleyi* cells. Samples were first vortexed vigorously to remove R11 cells from *E. huxleyi* and reduce bacterial cell clumping. Then a dilution series was prepared in L1-Si media, plated on ½ marine agar and incubated for 2 days at 30°C.

### Caspase-Like Activity Measurement

*In vitro* caspase-like IETDase (Ile-Glu-Thr-Asp) activity in *E. huxleyi* was measured as previously described by Bidle et al. (2007) with a few amendments. A single replicate was randomly chosen and analyzed for each experimental day. Briefly, aliquots of 850 μL of control cultures and co-cultures were pelleted by centrifugation for 10 min at 14,000 × *g* at 4°C. The supernatant was removed, discarded and replaced with 0.1 mL sterile PBS. The tube containing the pellet and PBS was immediately flash-frozen in liquid nitrogen, and stored at -80°C until processed. Pellets were resuspended and a subsample was analyzed for protein content using standard protein extraction (BCA protein assay, Pierce). The remaining sample was centrifuged and pellets were resuspended in caspase activity buffer according to manufacturer specifications (Caspase Activity Kit, EMB Millipore) and sonicated on ice. Cellular debris was pelleted (16,000 × *g*, room temp, 2 min) and cell lysates were incubated with IETD-AFC (Ile-Glu-Thr-Asp-7-amino-4-trifluoromethylcoumarin) according to the manufacturer’s instructions. Extracts were then incubated for 4 h at 25°C as IETDase activity from the cell lysate produces a fluorescent product from cleavage of ETD-AFC. Fluorescence was measured every 10 min using a Spectra Max Gemini XS plate reader (excitation 400 nm, emission 505 nm). Caspase activity was successfully abolished (>90%) with the irreversible caspase inhibitor z-VAD-FMK (z-Val-Ala-Asp-fluoromethyl-ketone; Calbiochem).

*In vivo* detection of active caspase-like proteases was accomplished using a cell permeable fluorescently labeled active site inhibitor (Millipore: FITC-VAD-FMK). Labeled algal proteases were visualized with epifluorescence microscopy after *in vivo* cell staining. After staining, cells were pelleted by centrifugation for 5 min at 5,000 × *g*, gently suspended in sterile PBS according to manufactures instructions; cells were imaged within 5 h of staining. Epifluorescence microscopy was used to assess algal chlorophyll autofluorescence. Cells were also stained with DAPI (4′,6-Diamidino-2-Phenylindole; Dihydrochloride; Life Technologies, Carlsbad, CA, USA) according to manufacturer’s instructions. DAPI was used instead of SYBRgreen-I, as the emission spectrum of the latter overlaps with that of the caspase inhibitor.

## Results

### Population Dynamics of *Emiliania huxleyi* and *Ruegeria* sp. R11 in Co-culture

Three cell types of *E. huxleyi* were tested for their interaction with R11 at 18 and at 25°C. For each algal strain tested, control cultures of the algae alone and *Ruegeria* sp. R11 alone, as well as a co-culture of R11 and algae, were prepared as previously described by Bramucci et al. (2015).

#### Coccolith-Bearing C Type *E. huxleyi*

**FIGURE 1:**
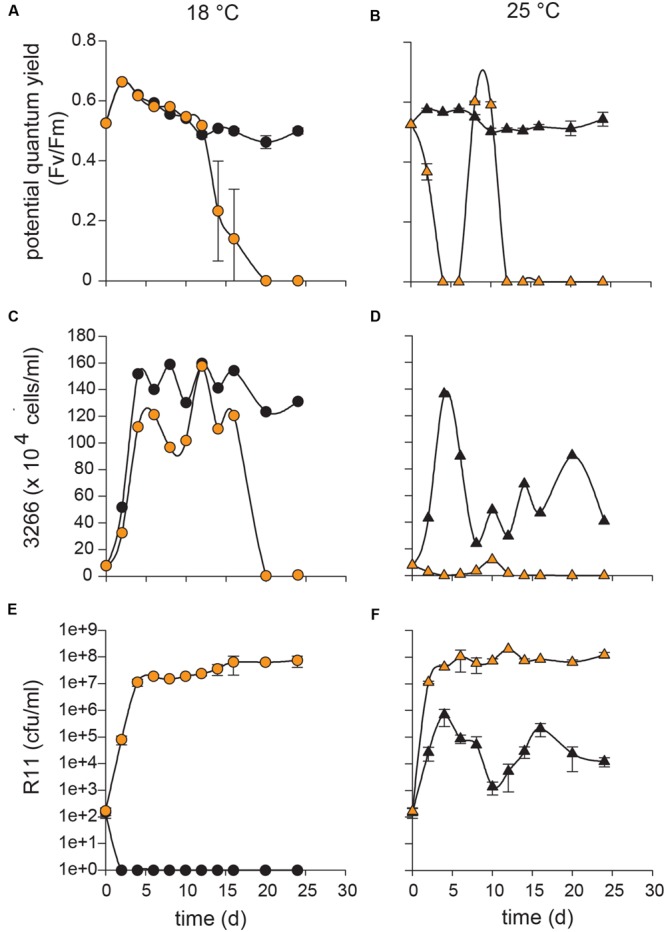
**Influence of temperature on co-cultures of *Ruegeria* sp. R11 with C type *Emiliania huxleyi* (CCMP3266).** R11 (10^2^ cells/ml) was co-cultured with CCMP3266 (10^5^ cells/ml) at 18 and 25°C and monitored over 24 days to determine the influence of temperature on co-cultures. For co-cultures and CCMP3266 grown alone, the potential quantum yield (*F*_v_/*F*_m_) was measured at 18°C **(A)** and 25°C **(B)**. Algal cell counts (cells/ml) were measured using flow cytometry for co-cultures and CCMP3266 alone at 18°C **(C)** and 25°C **(D)**. Bacterial enumeration (cfu/ml) was performed for R11 alone and in co-culture with CCMP3266 in L1-Si medium at 18°C **(E)** and 25°C **(F)**. Data for R11-CCMP3266 co-cultures are indicated with orange data points and control cultures (R11 or CCMP3266 grown alone) with black data points. All control cultures and co-cultures were performed in triplicate. Error bars: ±SE.

At 18°C, C type *E. huxleyi* (CCMP3266) in co-culture with R11 remained healthy until 14 days, when death of CCMP3266 was observed (**Figures 1A,C**). The PSII potential quantum yield (*F*_v_/*F*_m_) – a measure of photosynthetic efficiency hereafter referred to as yield (Schreiber, 1998) – of CCMP3266 in co-culture began to drop from 12 to 14 days, and continued dropping until the damage to PSII resulted in an undetectable yield at 20 days, and did not recover at any subsequent time in the experiment (**Figure 1A**). Algal cell numbers followed a similar pattern, with a small decrease occurring from 12 to 14 days, and a greater decrease to near zero values between 16 and 20 days (**Figure 1C**). In contrast, control cultures of CCMP3266 at 18°C retained a consistently high yield and cell density throughout the experiment (**Figures 1A,C**).

Death was observed much earlier in the co-culture of CCMP3266 with R11 grown at 25°C compared to 18°C (**Figures 1B,D**). At 25°C, the yield (**Figure 1B**) and algal cell density (**Figure 1D**) began to decline by 2 days and reached an undetectable level by 4 days (compared to 14 and 20 days at 18°C). A small resurgence in cell density with high yield values was observed on 8 days, which was again undetectable by 12 days and remained so through the experiment (**Figures 1B,D**). Like those grown at 18°C, control cultures of CCMP3266 at 25°C retained a consistently high yield throughout the experiment (**Figure 1B**). CCMP3266 cell density in control culture at 25°C was initially similar to the control culture at 18°C (1.4 × 10^6^ cells/mL and 1.5 × 10^6^ cells/mL, respectively, on 4 days), but later decreased on 6 and 8 days to approximately half of it’s peak cell density and then experienced large oscillations around this number from 10 to 24 days (**Figure 1D**).

The R11 population density in co-culture with CCMP3266 at 18 and 25°C both increased from 10^2^ cfu/mL to 10^7^ cfu/mL (**Figures 1E,F**). However, the 25°C co-cultures reached this cell density faster (on 2 days) than the co-culture at 18°C (on 4 days; **Figures 1E,F**). At both temperatures, the R11 populations benefited from the presence of CCMP3266. At 18°C, control R11 (bacteria alone) population density crashed by 2 days (**Figure 1E**), while at 25°C, the R11 population remained present, but experienced large oscillations from 10^3^ to 10^6^ cfu/mL for the remainder of the experiment (**Figure 1F**). The R11 control population remained present at a significant level throughout the experiment at 25°C but not 18°C, a temperature it grows well at in ½ marine broth, suggests that R11 does not thrive in L1-Si media at 18°C due to the additive effects of a low nutrient medium and low temperature.

#### Scale-Bearing Swarming S Type *E. huxleyi*

**FIGURE 2:**
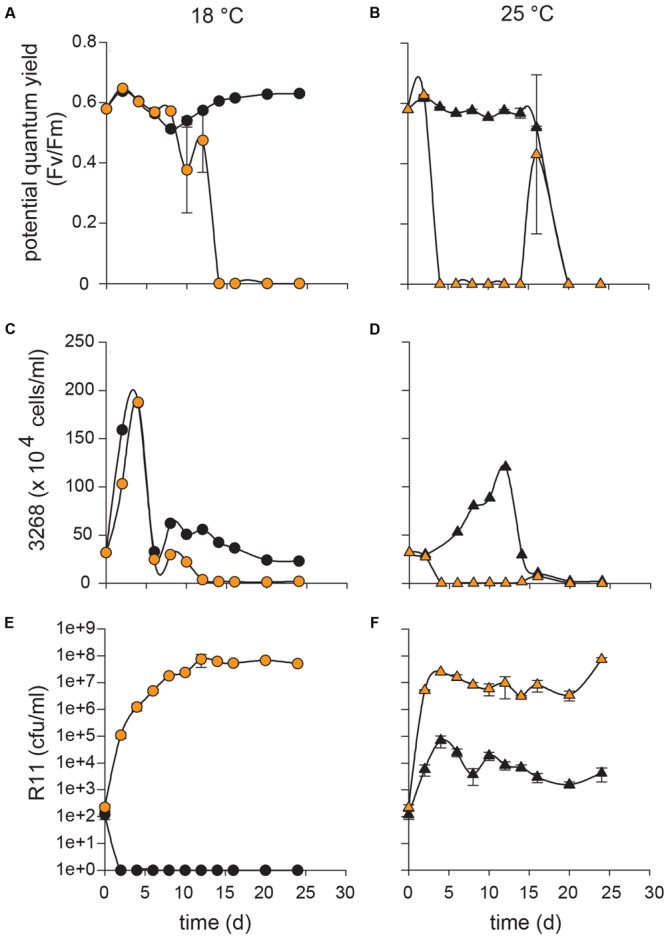
**Influence of temperature on co-cultures of *Ruegeria* sp. R11 with S type *Emiliania huxleyi* (CCMP3268).** R11 (10^2^ cells/ml) was co-cultured with CCMP3268 (10^5^ cells/ml) at 18 and 25°C and monitored over 24 days to determine the influence of temperature on co-cultures. For co-cultures and CCMP3268 grown alone, the potential quantum yield (*F*_v_/*F*_m_) was measured at 18°C **(A)** and 25°C **(B)**. Algal cell counts (cells/ml) were measured using flow cytometry for co-cultures and CCMP3268 alone at 18°C **(C)** and 25°C **(D)**. Bacterial enumeration (cfu/ml) was performed for R11 alone and in co-culture with CCMP3268 in L1-Si medium at 18°C **(E)** and 25°C **(F)**. Data for R11-CCMP3268 co-cultures are indicated with orange data points and control cultures (R11 or CCMP3268 grown alone) with black data points. All control cultures and co-cultures were performed in triplicate. Error bars: ±SE.

Similar to the C cell type (CCMP3266), the co-culture of the S cell type (CCMP3268) with R11 at 18°C remained healthy until 10 days, after which death was observed (**Figures 2A,C**). The yield began to decline on 10 days, becoming negligible by 14 days and no recovery was observed by 24 days (**Figure 2A**). Both control (CCMP3268 alone) and co-culture S cell density experienced a rapid increase from 0 to 4 days, but declined after 6 days (**Figure 2C**). The CCMP3268 control culture cell density remained steady at this lower level for the remainder of the experiment (**Figure 2C**). However, the algal cell density of the co-culture kept declining, approaching zero by 12 days, where it remained until the end of the experiment (**Figure 2C**).

Death was observed much earlier in the co-culture of CCMP3268 with R11 at 25°C in comparison to 18°C (**Figures 2B,D**). At this higher temperature, the yield (**Figure 2B**) and cell count (**Figure 2D**) of the co-culture were similar to control values (no R11) on 2 days, but had crashed by 4 days. Algal cell density remained near zero for the remainder of the experiment (**Figure 2D**). Co-culture yield values also remained undetectable, except for an anomaly on 16 days where a single replicate gave a detectable reading (**Figure 2B**). This type of outlier is due to the nature of the sacrificial sampling method and was observed in replicate experiments (results not shown). On a given sampling day, three replicates of the 1 mL wells are sacrificed and sampled for each culture type (algal control, bacterial control, and co-culture). At 25°C, control cultures of CCMP3268 retained a high yield for 10 days after the death of the co-culture, falling significantly only on 16 days (**Figure 2B**). Compared to the control culture at 18°C, which grew to ∼1.5 × 10^6^ cells/mL by 4 days and maintained this cell density throughout the experiment, the cell density of the CCMP3268 control culture at 25°C increased slowly, peaking at ∼1.3 × 10^6^ cells/mL on 12 days, after which it followed the same pattern as the yield (**Figure 2D**). Neither the yield, nor the cell count recovered by the end of the experiment.

R11 populations attained equally high density (10^7^ cfu/mL) in co-culture with CCMP3268 at both 18 and 25°C (**Figures 2E,F**). Similar to the co-culture with CCMP3266, this level was achieved twice as quickly at 25 as at 18°C (**Figures 2E,F**). Control populations of R11 reached the same density as control bacterial populations from the CCMP3266 co-culture experiment, crashing rapidly at 18°C (on 2 days) and maintaining their population at 25°C at a lower level than in the co-culture (**Figures 2E,F**).

#### Bald N Type *E. huxleyi*

**FIGURE 3:**
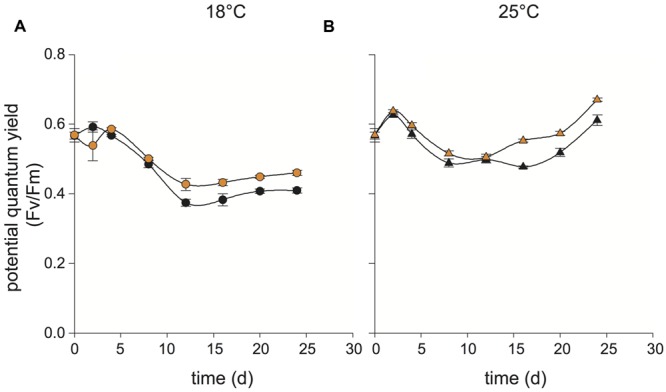
**Influence of temperature on co-cultures of *Ruegeria* sp. R11 with N type *Emiliania huxleyi* (CCMP2090).** R11 (10^2^ cells/ml) was co-cultured with CCMP2090 at 18 and 25°C and monitored over 24 days to determine the influence of temperature on co-cultures. For co-cultures and CCM2090 grown alone, the potential quantum yield (*F*_v_/*F*_m_) was measured at 18°C **(A)** and 25°C **(B)**. Data for R11-CCMP2090 co-cultures are indicated with orange data points and control cultures (CCMP2090 grown alone) with black data points. All control cultures and co-cultures were performed in triplicate. Error bars: ±SE.

At 18 and 25°C, both the co-culture and control culture of the N type *E. huxleyi* cells (CCMP2090) retained a high yield through 24 days (**Figures 3A,B**). Death was never observed in these co-cultures. Co-cultures were established with the same density of R11 (10^2^ cfu/mL) and reached the same population density (10^7^ cfu/mL) as it did in co-culture with CCMP3266 and CCMP3268. Absolute numbers of R11 were not quantified at every time point within the experiment, but its presence was confirmed with drop plating of the co-culture on ½ marine agar at every sampling point. Flow cytometry was not run for this cell type, as no effect of co-culturing on yield or minimum fluorescence (a proxy for chlorophyll fluorescence) was observed.

### Observation of Algal Bleaching in *E. huxleyi* and *Ruegeria* sp. R11 Co-Cultures

**FIGURE 4:**
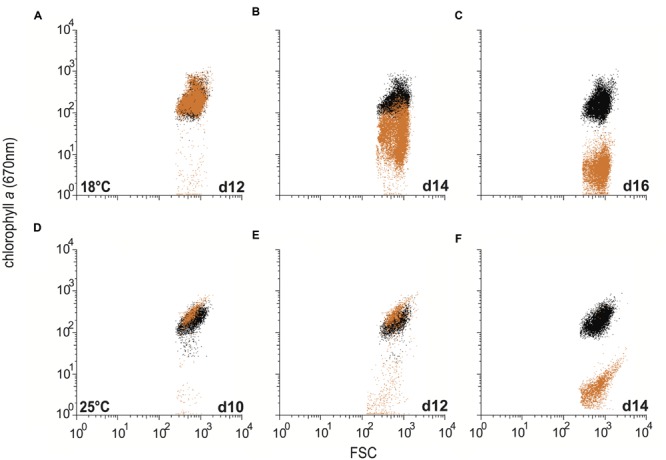
**Bleaching in C type *Emiliania huxleyi* (CCMP3266) in co-culture with *Ruegeria* sp. R11.** Control cultures of CCMP3266 and co-cultures of CCMP3266 with R11 were assessed for chlorophyll content using flow cytometry at 18°C on 12 days **(A)**, 14 days **(B)**, and 16 days **(C)**; and at 25°C on 10 days **(D)**, day 12 **(E)**, and day 14 **(F).** Data for R11-CCMP3266 co-cultures are indicated with orange data points and control cultures (CCMP3266 grown alone) with black data points.

**FIGURE 5:**
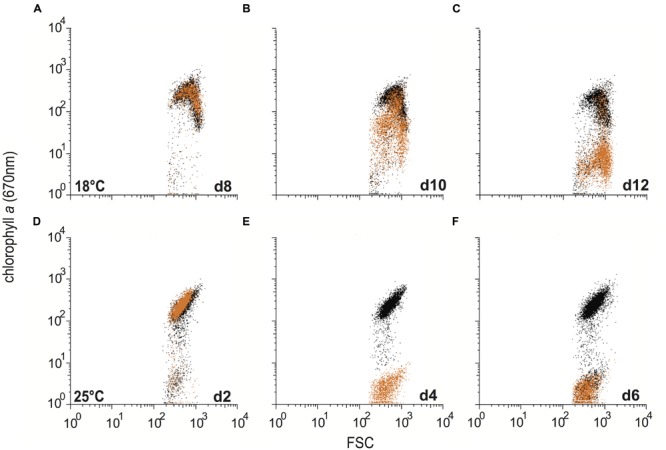
**Bleaching in S type *Emiliania huxleyi* (CCMP3268) in co-culture with *Ruegeria* sp. R11.** Control cultures of CCMP3268 and co-cultures of CCMP3268 with R11 were assessed for chlorophyll content using flow cytometry at 18°C on 8 days **(A)**, 10 days **(B)**, and 12 days **(C)**; and at 25°C on 2 days **(D)**, 4 days **(E)**, and 6 days **(F)**. Data for R11-CCMP3268 co-cultures are indicated with orange data points and control cultures (CCMP3268 grown alone) with black data points.

Since R11 is known to cause bleaching in the macroalga *D. pulchra* (Case et al., 2011), the bleaching effect of R11 on *E. huxleyi* was assessed using flow cytometry. R11 pathogenesis of *E. huxleyi* caused the loss of chlorophyll autofluorescence, or bleaching, of CCMP3266 (**Figure 4**) and CCMP3268 (**Figure 5**). At 18°C, co-culture populations of CCMP3266 and CCMP3268 with R11 are indistinguishable from control populations (algae alone) at 12 and 8 days respectively, when the chlorophyll autofluorescence (670 nm) of cells was plotted against the forward scatter for both populations (**Figures 4A** and **5A**). On 14 and 10 days, when yield and cell density indicated the start of algal decline (**Figures 1A,C** and **2A,C**), CCMP3266 and CCMP3268 cells lost chlorophyll autofluorescence, but retained forward scatter values (**Figures 4B** and **5B**). This shows that the algae lose chlorophyll autofluorescence before cell size (i.e., lysis). This decrease in chlorophyll autofluorescence happened relatively gradually (compared to 25°C), resulting in a ‘smear’ of cells in the process of losing chlorophyll on the scatter plot. On days where yield and cell density were near zero, a population of cells was present with the same forward scatter as control cultures, but almost all fluorescence (chlorophyll) was gone (**Figures 4C** and **5C**).

At 25°C, the decrease in chlorophyll content occurred more rapidly, and the gradual loss observed at 18°C is not present (**Figures 4** and **5**). Instead, the co-cultures appear to experience a rapid loss of fluorescence (chlorophyll) from populations similar to control cultures with no bacteria (same forward scatter and chlorophyll values), to populations of cells with the same forward scatter as control cultures, but a low level of fluorescence (chlorophyll) by the next time point (**Figures 4** and **5**).

As a secondary measure of cell death, cells were stained cells with SYBRgreen-I that stains DNA. The DNA content of cells decreased in both CCMP3266 (Supplementary Figure S1) and CCMP 3268 following the same general pattern as the chlorophyll bleaching. However, this process of DNA loss was slower and cells were observed microscopically to have DNA throughout the cytoplasm when co-cultured with R11, while control cells had a clear nucleus (Supplementary Figure S1).

### Measurement of Caspase-Like Activity in C Type *E. huxleyi* Co-Cultures with *Ruegeria* sp. R11

**FIGURE 6:**
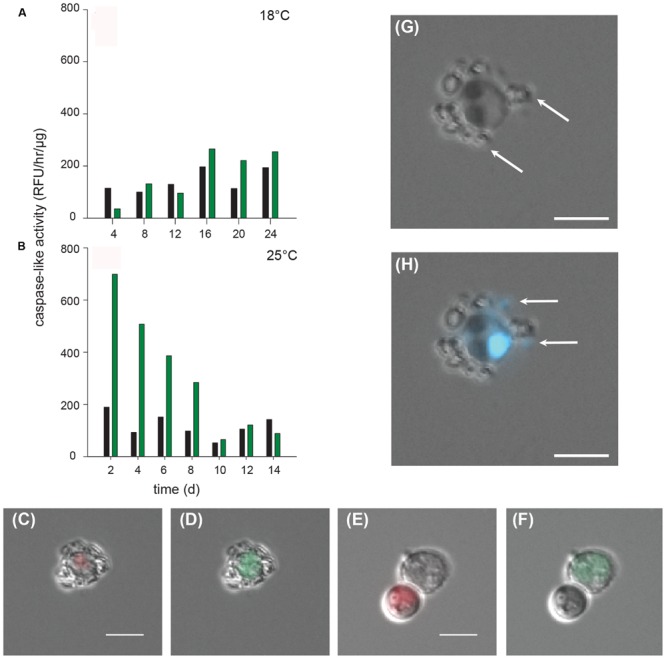
**Detection of caspase-like activity in C type *Emiliania huxleyi* (CCMP3266) in co-culture with *Ruegeria* sp. R11.** R11 (10^2^ cells/ml) was co-cultured with CCMP3266 (10^5^ cells/ml) at 18 and 25°C to determine the influence of temperature on the degree of IETDase activity detected in CCMP3266 throughout the co-culture with R11. *In vitro* IETDase activity was measured (Caspase Activity Kit, EMB Millipore) at 18°C for CCMP3266 grown alone and in co-culture with R11 **(A)**; and at 25°C for CCMP3266 grown alone and in co-culture with R11 **(B)**. Data for R11-CCMP3266 co-cultures are indicated with green bars and control cultures (CCMP3266 grown alone) with black bars. Chlorophyll autofluorescence (red) was monitored microscopically in CCMP3266 cells at 18°C **(C)** and 25°C **(E)**. *In vivo* detection of active capsase-like molecules (green) was monitored microscopically in cells stained with CaspACE (*in situ* VAD-marker: FITC-VAD-FMK) at 18°C **(D)** and at 25°C **(F)**. DIC imaging shows cells bearing coccoliths (individual coccoliths indicated with ‘Co’) and DNA within algal and bacterial cells is stained with DAPI **(G)** and attached R11 cells are distinguished from coccoliths as bacteria contain DAPI stained DNA (bacteria indicated with ‘R11’) **(H)**. The scale bar is 5 μm.

The major known pathogens of *E. huxleyi* are viruses, which are collectively called *E. huxleyi* viruses (EhVs). Infection by these viruses triggers caspase-like activity (likely mediated by algal metacaspase activity), which coincides with death and production of viral particles in various diploid N type *E. huxleyi* strains (Bidle et al., 2007). Caspase-like activity has been observed in cells after the loss of their chlorophyll and therefore of the corresponding autofluorescence (Bidle et al., 2007). To determine if a bacterial pathogen of *E. huxleyi* would also trigger caspase-like activity, CCMP3266 was assayed for caspase-like IETDase (Ile-Glu-Thr-Asp) using the Caspase-8 Activity Kit (EMB Millipore). In the control cultures (*E. huxleyi* only) at 18 and 25°C, a background level of caspase-like activity was present throughout the experiment (∼50–200 RFU/h/μg; **Figures 6A,B**). For CCMP3266 in co-culture with R11 at 18°C, there was a slight (0.25- to 0.5-fold) increase in caspase-like activity on 16, 20, and 24 days (time points at which the co-culture was dead or dying), compared to the control (**Figure 6A**). However, the caspase-like activity was a much greater in the co-culture at 25°C – a 3.5-fold increase was observed at 2 and 4 days, and a twofold increase on 6 and 8 days (all days on which the co-culture was dead or dying; **Figure 6B**).

Caspase-like activity was directly visualized using microscopy of cells stained with a fluorescently labeled caspase-marker (FITC-VAD-FMK). Cells showing clear VAD labeling - an indicator of active caspase-like proteases - in both the 18 and 25°C R11-CCMP3266 co-cultures were characterized as having less or no chlorophyll and compromised cell integrity. The image in **Figures 6C,D** is a representative cell taken from an 18°C R11-CCMP3266 co-culture at 14 days. This cell bears coccoliths, shows minimal autofluorescence (indicating a small amount of chlorophyll; **Figure 6C**) and displays caspase-like activity (**Figure 6D**). On the other hand, chlorophyll-containing cells (showing strong autofluorescence) were rare in declining R11-CCMP3266 co-cultures. These were typical in young control cultures, however, some could be found even within the 25°C co-culture using microscopy (Supplementary Figure S2). In addition to significant chlorophyll autofluorescence, these cells were characterized by the presence of structural integrity and lack of visible FITC-VAD-FMK labeling. The image in **Figures 6E,F** was taken from 4 days R11-CCMP3266 co-culture at 25°C and displays a chlorophyll-containing cell (smaller and showing chlorophyll autofluorescence in red but lacking FITC-VAD-FMK labeling) and a cell displaying strong caspase activity (larger and staining green from FITC-VAD-FMK labeling but lacking autofluorescence from loss of chlorophyll). In this image, both cells have lost their coccoliths, a normal result of senescence (Chow et al., 2015). In many cases, R11 cells were observed attached to *E. huxleyi* cells (**Figures 6G,H**).

## Discussion

### *Ruegeria* sp. R11 Pathogenicity Varies between Cell Types of *Emiliania huxleyi*

The present study demonstrates that *Ruegeria* sp. R11 is a pathogen of *E. huxleyi*, but that this pathogenicity, or host resistance, is strain dependent. Both *E. huxleyi* CCMP3266 and CCMP3268 are killed when in co-culture with R11 (**Figures 1** and **2**, respectively). CCMP3268, an S type haploid flagellated cell, was originally isolated from cultures of CCMP3266, a C type diploid coccolith bearing cell isolated from the Tasman Sea, after part of the original culture of CCMP3266 was observed to undergo a shift to this haploid cell type (Frada et al., 2008; von Dassow et al., 2009). It is hypothesized that CCMP3268 is the sexual cell of CCMP3266. However, since neither meiosis nor syngamy has ever been directly observed, this cannot be confirmed. Given this relationship between CCMP3266 and CCMP3268, it follows that they should be similar in their sensitivity to pathogens unless the cell type conveyed resistance. Transcriptomic analyses have shown that these strains display ∼50% transcript similarity, with the major functional differences relating to motility and biogenic CaCO_3_ production – the haploid CCMP3268 cells are flagellated while the diploid CCMP3266 cells are coccolith bearing (von Dassow et al., 2009). However, it has been shown that while CCMP3266 is sensitive to the bloom-collapse causing EhVs, CCMP3268 is resistant due to a lack of host recognition by the virus (Frada et al., 2008). As opposed to the C or S type strains, bald N type *E. huxleyi* CCMP2090 is seemingly resistant to infection by R11 (**Figure 3**), although interestingly, CCMP2090 is susceptible to two of the major strains of EhV (EhV1 and EhV86; Fulton et al., 2014). Since the bacteria infects different cell types than the previously described EhVs, the complex interplay between these two algal pathogens will be important to understand as the ecology of *E. huxleyi* blooms.

It is unclear from the present study what is the key difference between the *E. huxleyi* strains that causes the observed variability in susceptibility to R11 infection, but there are several possibilities. CCMP2090 is the diploid axenic non-coccolith bearing (bald) isolate of the diploid coccolith bearing CCMP1516, which was collected off the coast of Ecuador, making it geographically distant from both CCMP3266 and R11, which were both isolated from the Tasman Sea. The ability of phylogenetically closely related strains of the Roseobacter clade to induce gall formation on species of *Prionitis* has been shown to be geographically specific – strains of gall-forming roseobacter isolated from infected *Prionitis* in one geographical area were unable to induce gall formation in closely related *Prionitis* from a distant geographical location (Ashen and Goff, 2000). Similarly, it has been demonstrated in land plants that geographically distant subpopulations of a single species can differ in their resistance/susceptibility to pathogens, likely due to decreased interbreeding (Thrall et al., 2001). Diploid cells of *E. huxleyi* have been shown to have a more complex transcriptome than haploid cells, suggesting that there may be greater phenotypic diversity between diploid strains (von Dassow et al., 2009) and thus greater diversity in their resistance to pathogens (i.e., CCMP3266 and CCMP2090 are both diploid strains). Additionally, the nature of the CCMP2090 cell – naked, lacking both coccoliths and organic scales – may contribute to the differences in sensitivity between strains. It may be that R11 has different levels of attachment and/or colonization of the naked, organic or calcite liths covering *E. huxleyi’s* cell surface.

N type cells, like CCMP2090, are thought to be a rare natural variant of C type cells, such as CCMP3266 (Paasche, 2002). However, if the mutation that causes the non-calcifying N cell type to occur provides an escape strategy from pathogens, the abundance and distribution patterns of this cell type may change in the future, and this would have consequences for the carbon sequestration role of *E. huxleyi*.

Although the mode of R11’s pathogenesis is not currently known, several virulence factors have been identified, including the production of ammonia (inhibits photosynthesis), cytolytic toxins (lyses cells; Fernandes et al., 2011), and glutathione peroxidase (resists oxidative bursts from the host; Gardiner et al., 2015). It has also been hypothesized that the virulence of R11 may be related to its production of indole-3-acetic acid (IAA) – a phytohormone with various roles in the growth and development of land plants known to be produced by R11 (Fernandes et al., 2011). An extracellular excess of IAA causes hypertrophy and may increase the amount of algal exudates available to R11 (Fernandes et al., 2011). Interestingly, it has recently been shown that the exogenous addition of IAA causes increased cell permeability and cell size in CCMP2090, but not in CCMP3266 (Labeeuw et al., 2016). However, a difference in R11 virulence toward CCMP3266 is observed when tryptophan is added to stimulate IAA production, R11 killing CCMP3266 twice as fast (1 day compared to 2 days without the addition of tryptophan; Labeeuw et al., 2016). This is suggestive that IAA influences the virulence of R11 toward *E. huxleyi*. However, this effect is likely influenced by host factors, given that unlike CCMP3266, CCMP2090 cell size and permeability are responsive to IAA, but is not susceptible to the virulence of R11.

### The Virulence of R11 toward *E. huxleyi* Is Temperature-Enhanced

The decrease in CCMP3266 and CCMP3268 health observed when grown in co-culture with R11 at 25°C compared to 18°C indicates that the pathogenicity of this bacterium toward them is temperature-enhanced. While R11 ultimately causes the death of CCMP3266 and CCMP3268 at both 18 and 25°C, the course of the infection is accelerated at elevated temperature (**Figures 1A–D** and **2A–D**). This increase in the pathogenicity of R11 at elevated temperature cannot be explained by differential bacterial loads, as the R11 populations reached the same order of magnitude (10^7^ cfu/mL) in co-culture with both algal strains at both temperatures (**Figures 1E,F** and **2E,F**). Although R11 attains this carrying capacity 2–4 days earlier at 25°C than at 18°C, this is also insufficient to explain the differences (**Figures 1E,F** and **2E,F**). With CCMP3266, the initial drop in algal yield at 25°C occurs on the same day that R11 cell density reach their carrying capacity, while at 18°C, algal death in co-culture does not begin until 10 days after the carrying capacity of R11 is reached (**Figure 1**). With CCMP3268, the timelines are slightly closer together, with the death of the 25°C co-culture beginning 2 days after R11 cell density reached carrying capacity and the death of the 18°C co-culture beginning 4 days after R11 carrying capacity had been reached (**Figure 2**).

The differences in timeline leading to death also cannot be explained by differences in the photosynthetic health of the algae at the two temperatures. CCMP3266 control cultures displayed equal yield values at both temperatures for the duration of the experiment (**Figures 1A,B**). CCMP3268 control cultures also maintained equivalent yield values at both 18 and 25°C, until the control culture experienced death starting on 16 days (**Figures 2A,B**).

Taken together, these results support the hypothesis that the virulence of R11 on *E. huxleyi* is temperature-enhanced. This is in keeping with the original *Delisea pulchra*-R11 model of virulence in which R11 was pathogenic to *D. pulchra* at 24°C, but not pathogenic at 19°C (Case et al., 2011). Temperature-enhanced bacterial pathogens have been linked to several other algal diseases including ‘white tip disease’ in *Gracilaria conferta* – in which a bacterial isolate was found to be the causative agent and that increasing temperature above 20°C increased the rate of infection (Weinberger et al., 1994). Another example of temperature-enhanced virulence in the marine environment can be found in the bleaching of the coral *Pocillopora damicornis* by the bacterium *Vibrio coralliilyticus*, triggered by elevated temperature (Kushmaro et al., 1996; Ben-Haim et al., 2003; Rosenberg et al., 2009). In fact, this temperature-induced bleaching results from an attack by *V. coralliilyticus* on the zooxanthellae algal symbionts living within the coral tissue (Ben-Haim et al., 2003). It appears that the increased pathogenicity in this case was due to both the increased expression of virulence factors and a possible increase in sensitivity of the algae to pathogen attack due to temperature stress (Kushmaro et al., 1996; Ben-Haim et al., 2003; Rosenberg et al., 2009).

In the present study, there is evidence of temperature stress in CCMP3266 and CCMP3268 at 25°C, as there were marked differences in the algal population size and dynamics at 18 and 25°C. For CCMP3266 at 25°C, the population size initially followed the same trajectory as the culture at 18°C, reaching nearly the same peak cell density, but subsequently dropping to around half the density of the 18°C culture, where it stabilized for the remainder of the experiment (**Figures 1C,D**). For CCMP3268, the control culture at 25°C followed a completely different trajectory to the 18°C control culture, slowly increasing to a peak only two thirds the density of the maximum at 18°C, 8 days later (**Figures 2C,D**). After this peak, cell density dropped sharply and remained near zero for the remainder of the experiment.

The fact that the cell densities were lower at 25°C for both CCMP3266 and CCMP3268 likely indicates temperature stress. The reported temperature range of *E. huxleyi* is highly variable (spanning 6–26°C; Rhodes et al., 1995; Paasche, 2002; Daniels et al., 2014), with marked differences in temperature optima reported even between strain clones (Paasche, 2002). In the present study, while cultures of both CCMP3266 and 3268 grow normally at 18°C (with a rapid log phase and a stable stationary phase), they both display altered dynamics at 25°C (slow initial growth rate and low or un-sustained stationary phase), which is near the upper limit of the species’ temperature range. However, 25°C is an ecologically relevant temperature for these strains, as current SST in the Tasman Sea, where both CCMP3266/3268 and R11 originate, regularly reaches 25°C in the austral summer. This area – sometimes referred to as the ‘Tasman Hot Spot’ – is predicted to have a rate of SST warming 3–4 times the global average (Oliver et al., 2014). Additionally, a metagenomic study has shown that EhVs are absent from populations of *E. huxleyi* in warm equatorial waters (von Dassow et al., 2015). This raises the possibility that regions likely to be even warmer in the future, such as the Tasman Sea, which currently host populations of *E. huxleyi* infected with EhVs, may soon represent a niche open to new pathogens such as R11.

It is unclear from the data presented whether the cause of the increase in pathogenicity of R11 at 25°C was the result of increased susceptibility of *E. huxleyi*, or was due to an increase in the production of virulence factors by R11 at elevated temperature, or a combination of the two factors. Plant pathogens are known to be triggered by temperatures outside the optimal range for host growth – in other words, by temperatures at which the defenses of the host may be compromised (Smirnova et al., 2001). For example, the blight pathogen *Pseudomonas syringae* significantly increases production of a phytotoxin at 18°C (7–10°C below the growth optimum of its host; Budde and Ullrich, 2000).

It is possible that R11 is an opportunistic pathogen, as its host range appears to be broad – including a red macroalga (Case et al., 2011) and a haptophyte (present study). For a pathogen with diverse hosts, a versatile strategy of triggering virulence might be to sense the stress of a host directly, instead of sensing the conditions that would cause a host’s defenses to be compromised. This is a mechanism known to exist in *Phaeobacter gallaeciensis* BS107, another member of the Roseobacter clade. *P. gallaeciensis* produces algaecides in response to *p*-coumaric acid (*p*CA) – produced by *E. huxleyi* and thought to be a product of senescence (Seyedsayamdost et al., 2011). However, R11 is unlikely to use this particular molecule as a cue, since the addition of *p*CA did not stimulate a change in its production of small molecules (Seyedsayamdost et al., 2011). The evidence from the present study – the fact that R11 displays a broad host range and increased virulence under conditions at which the host displays evidence of temperature stress – supports the hypothesis that R11 is an opportunistic pathogen.

### *Ruegeria* sp. R11 Causes Bleaching in *E. huxleyi*

Bleaching – the loss of pigmentation – is a common phenomenon in marine corals and macroalgae (Jenkins et al., 1999; Douglas, 2003; Egan et al., 2013). In corals, this color loss refers to the death or loss of the symbiotic algae that live within the coral’s tissue – a temperature-dependent effect often linked to bacterial infection – that ultimately leads to the death of coral host (Kushmaro et al., 1996; Ben-Haim et al., 2003; Rosenberg et al., 2009). In macroalgae, the bleaching effect is due to the degradation of photosynthetic pigment that, depending of the extent of the bleaching, may lead to the death of the whole organism. In the present study, a color change was clearly visible in dead or dying cultures of *E. huxleyi*. R11-*E. huxleyi* co-culture wells changed from green to white. This bleaching was also evident from the flow cytometry results (**Figures 4** and **5**). In the case of both CCMP3266 (**Figure 4**) and CCMP3268 (**Figure 5**), during culture death, cells maintained their size (forward scatter) but lost their chlorophyll α content over 2 days at 25°C (**Figures 4D–F** and **5D–F**) or 4 days at 18°C (**Figures 4A–C** and **5A–C**).

Algal bleaching has been mostly attributed to temperature or UV stress alone (Jenkins et al., 1999), except in the case of *D. pulchra*, in which R11 is the temperature-dependent causative agent of the bleaching disease – *D. pulchra* grown without R11 at high temperature does not exhibit bleaching (Case et al., 2011). With the mentioned exception of *D. pulchra*, these studies do not assess the microbial community component of the system, and as such, bacterially mediated temperature induced bleaching in marine algae could be far more common than the literature reports. Here we demonstrate that it occurs in a microscopic unicellular haptophyte, distantly related, both phylogenetically and physiologically, to the red macroalgae in which it was previously found.

### Increased Caspase-Like Activity in Co-cultures of *E. huxleyi* and *Ruegeria* sp. R11

While PCD was historically considered a phenomenon linked to multicellularity, it has recently been identified in several unicellular lineages, including coccolithophores (Bidle et al., 2007; Bidle and Kwityn, 2012). PCD is the genetically programmed deconstruction of cellular components by highly specific proteases. Caspases are cysteine proteases that cleave target substrate proteins containing the corresponding cleavage motif (4–5 amino acid motifs), resulting in apoptotic-PCD. While metacaspases differ from true caspases in both activity and specificity, there are reports of target substrates being similar between these enzymes (Sundström et al., 2009), as well as some reports of metacaspases exhibiting a biochemical activity (e.g., cleavage motifs) that is similar to that of true caspases (Madeo et al., 2002; Wilkinson and Ramsdale, 2011). Caspase-like activity was observed both *in vivo* and *in vitro* in the co-culture of CCMP3266 and R11. The method used in this study to quantify caspase-like activity *in vitro* detects the activity of IETDase caspases and structurally similar caspase-like proteases, some of which have been linked to increased metacaspase gene expression in the model EhV system (Bidle et al., 2007). Since the genome of *E. huxleyi* does not contain the genes encoding for caspases, but does contain 13 genes encoding for metacaspases (Choi and Berges, 2013), the activity detected here is likely due to the latter. Some background level of caspase-like activity was found in control cultures of CCMP3266 alone at both temperatures (**Figures 6A,D**), which has been previously reported in the viral experiments (Bidle et al., 2007; Bidle and Kwityn, 2012). A slight increase in activity from that background level was detected in co-cultures on days during which CCMP3266 was dead or dying at 18°C, and a large increase on days during which CCMP3266 was dead or dying at 25°C. This spike in caspase-like activity at 25°C might explain why death happened much earlier and more rapidly at elevated temperature. Bacterial pathogens of several land plant genera, including *Solanum* and *Arabidopsis*, have been shown to trigger the expression of metacaspases during the course of infection (Coll et al., 2011), but to our knowledge, caspase-like activity has not previously been shown to be induced in marine algae by a bacterial pathogen.

## Conclusion

Natural blooms of *E. huxleyi* often experience a rapid collapse that has been attributed to lytic EhV infections causing PCD in the blooming algae (Vardi et al., 2009). However, it has recently been demonstrated that EhV strains become avirulent at increased temperature due to a change in the structure of the glycosphingolipid required for viral recognition (Kendrick et al., 2014). This algal resistance is gained with only a 3°C increase in temperature – from 18 to 21°C (Kendrick et al., 2014).

In the context of a rapidly warming ocean, the emergence of temperature-induced resistance in *E. huxleyi* to its major pathogen may present an ecological gap. Our findings indicate that opportunistic bacterial pathogens like R11 with temperature-enhanced virulence have the ability to fill this gap and a transition between viral and bacterial disease outbreaks in *E. huxleyi* may be observed as SST continues to rise.

## Author Contributions

TM and RC conceived the research. AB, TM, and RC conducted the flow cytometry, TM, AB, and KY conducted the growth experiments. TM, AB, and RC drafted the manuscript. All authors have read and approved the manuscript.

## Conflict of Interest Statement

The authors declare that the research was conducted in the absence of any commercial or financial relationships that could be construed as a potential conflict of interest.
