# Supplementary figures and images for: A Bacterial Pathogen Displaying Temperature-Enhanced Virulence of the Microalga Emiliania huxleyi
Source: Front Microbiol. 2016 Jun 13;7:892. doi: 10.3389/fmicb.2016.00892 (PMC4904034; doi:10.3389/fmicb.2016.00892)

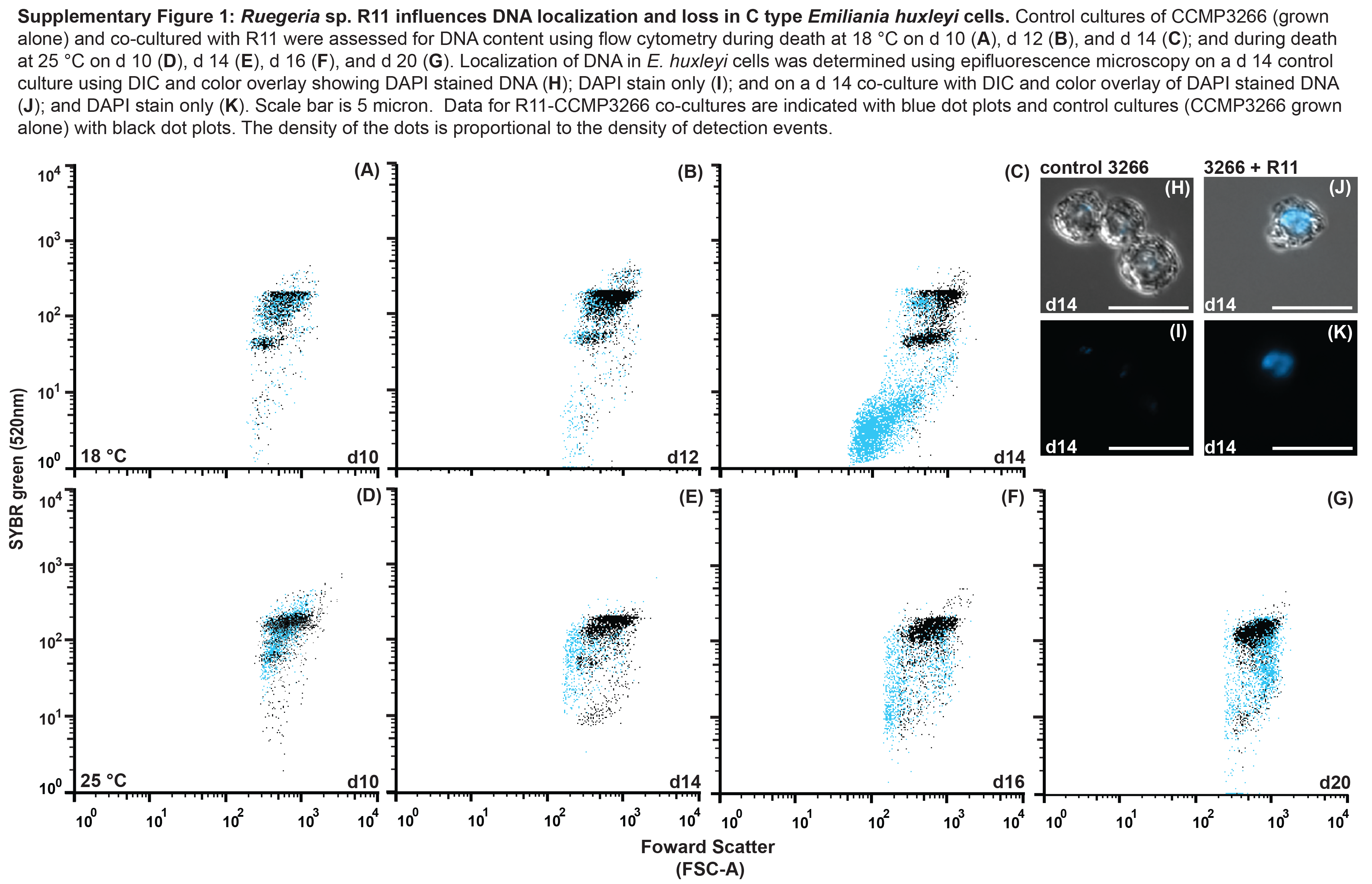

Supplement: Supplementary file 1 [file Image_1.TIF]

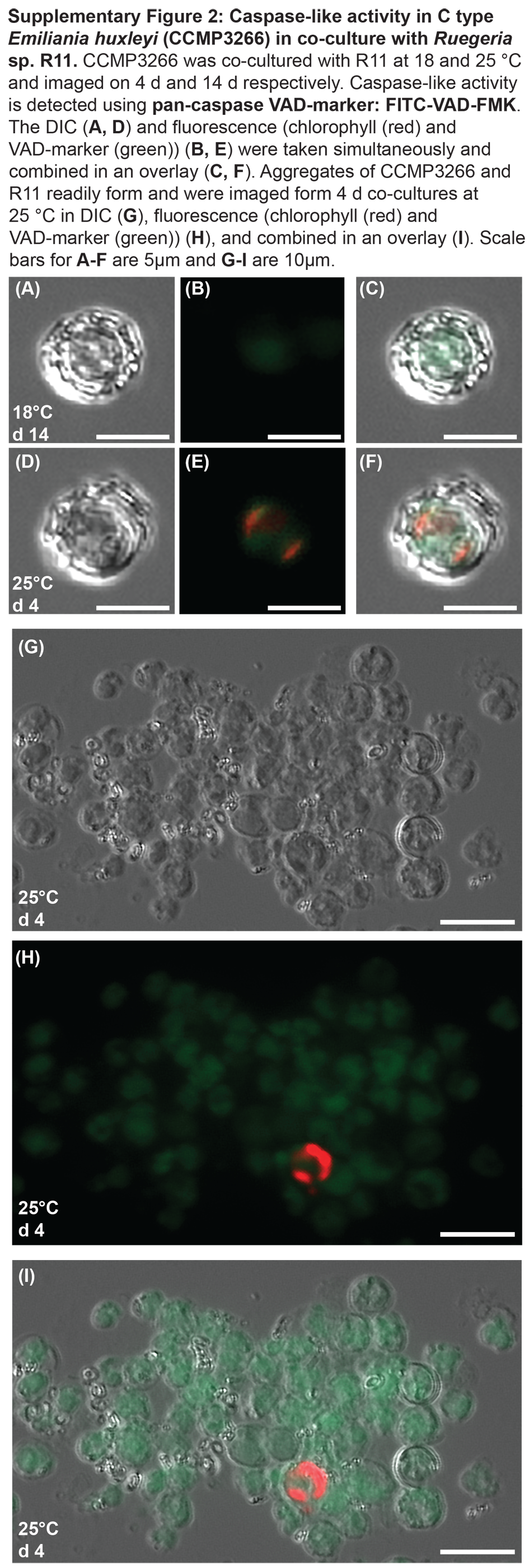

Supplement: Supplementary file 2 [file Image_2.TIF]
